# Identification of a nutrient-sensing transcriptional network in monocytes by using inbred rat models on a cafeteria diet

**DOI:** 10.1242/dmm.025528

**Published:** 2016-10-01

**Authors:** Neus Martínez-Micaelo, Noemi González-Abuín, Ximena Terra, Ana Ardévol, Montserrat Pinent, Enrico Petretto, Jacques Behmoaras, Mayte Blay

**Affiliations:** 1 Mobiofood Research Group, Department of Biochemistry and Biotechnology, Universitat Rovira i Virgili, 43003 Tarragona, Spain; 2 MRC Clinical Sciences Centre, Imperial College London, Hammersmith Hospital, Du Cane Road, London W12 0NN, UK; 3 Duke-NUS Graduate Medical School Singapore, 8 College Road, Singapore 169857, Republic of Singapore; 4 Centre of Complement and Inflammation Research, Imperial College London, Du Cane Road, London W12 0NN, UK

**Keywords:** Cafeteria diet, Inbred rats, Monocyte transcriptome, LEW, WKY

## Abstract

Obesity has reached pandemic levels worldwide. The current models of diet-induced obesity in rodents use predominantly high-fat based diets that do not take into account the consumption of variety of highly palatable, energy-dense foods that are prevalent in Western society. We and others have shown that the cafeteria (CAF) diet is a robust and reproducible model of human metabolic syndrome with tissue inflammation in the rat. We have previously shown that inbred rat strains such as Wistar Kyoto (WKY) and Lewis (LEW) show different susceptibilities to CAF diets with distinct metabolic and morphometric profiles. Here, we show a difference in plasma MCP-1 levels and investigate the effect of the CAF diet on peripheral blood monocyte transcriptome, as powerful stress-sensing immune cells, in WKY and LEW rats. We found that 75.5% of the differentially expressed transcripts under the CAF diet were upregulated in WKY rats and were functionally related to the activation of the immune response. Using a gene co-expression network constructed from the genes differentially expressed between CAF diet-fed LEW and WKY rats, we identified acyl-CoA synthetase short-chain family member 2 (*Acss2*) as a hub gene for a nutrient-sensing cluster of transcripts in monocytes. The *Acss2* genomic region is significantly enriched for previously established metabolism quantitative trait loci in the rat. Notably, monocyte expression levels of *Acss2* significantly correlated with plasma glucose, triglyceride, leptin and non-esterified fatty acid (NEFA) levels as well as morphometric measurements such as body weight and the total fat following feeding with the CAF diet in the rat. These results show the importance of the genetic background in nutritional genomics and identify inbred rat strains as potential models for CAF-diet-induced obesity.

## Abstract (precis)

**Summary:** Feeding with a cafeteria diet (CAF) is a reproducible model of human metabolic syndrome in the rat. By using inbred rat models of nutrigenomics, we have studied the effect of CAF on monocyte transcriptome.

## INTRODUCTION

Obesity represents an important health problem in human populations and there is need for new animal models that mimic optimally the main characteristics of human obesity.

Susceptibility to obesity has a large underlying genetic component with modulatory environmental factors such as diet (Barsh et al., 2000; Bouchard, 2008; Hetherington and Cecil, 2009; Ordovas, 2008; Pérusse and Bouchard, 2000; Speakman, 2004). The administration of cafeteria (CAF) diet to rats is considered as a robust model of the human metabolic syndrome and its related pathologies, and has been widely used to understand the genetic factors that underlie obesity. Feeding with a CAF diet leads to obesity due to the continuous hyperphagia as a result of the voluntary intake of highly palatable and energy-dense cafeteria-style foods, which are typically found in Western-type diets (Sampey et al., 2011).

Importantly, the genetic background of the selected strain is an important determinant of the phenotypic diversity observed in diet-induced obesity models. Hence, by comparing the responses of Lewis (LEW) and Wistar Kyoto (WKY) rats to an obesogenic diet, we recently showed significant differences in metabolic and morphometric parameters (Martínez-Micaelo et al., 2016). Specifically, our results suggested the leptin signalling pathway as a divergent point between the strain-specific adaptations to CAF diet. LEW rats display a typical obesity phenotype, characterised by an increase in body weight, adiposity and dyslipidaemic and hyperglycaemic profiles, accompanied by a preferential metabolisation of carbohydrates, as opposed to WKY rats, which preferentially metabolised lipids. Despite their body weight and adiposity gain, WKY rats maintained metabolic homeostasis as a result of the obesogenic diet showing a differential regulation in the leptin axis.

In obesity, the prolonged over-nutrition is characterised by the restrained ability of the adipose tissue to store energy as fat. In parallel, a cytokine-driven response characterised by the secretion of pro-inflammatory chemokines underlie obesity-induced inflammatory reactions. Among others, monocyte chemotactic protein-1 (MCP-1, also known as CCL2) is one of the most important cytokines due to its ability to recruit peripheral monocytes to the adipose tissue (Iyer et al., 2010; Osborn and Olefsky, 2012). Hence, the continuous activation of the local inflammatory response triggered by obesity could result in the widespread activation of the immune system (Lumeng and Saltiel, 2011).

Peripheral blood monocytes are powerful stress-sensing immune cells that play central roles in the regulation of innate immune responses through the release of inflammatory cytokines and the activation of the adaptive immunity (Parihar et al., 2010). Monocytes are of particular interest because, as circulating immune cells, they are exposed to the systemic environment, including the metabolic factors and pro-inflammatory cytokines produced and secreted by tissues and organs. Thus, the regulation of monocyte gene expression profile might reflect the physiological state of the whole organism.

Here, we studied the CAF-diet-induced modulation of peripheral monocyte transcriptome from inbred LEW and WKY rats. Considering the isogenic and homozygous nature of these inbred strains and using the CAF diet as an inducer of obesity, both the genetic background and environmental factors can be controlled, providing a powerful tool for understanding the ‘gene–diet’ interactions that underlie the development of complex traits such as obesity. These inbred strains have been widely studied for their differential susceptibility to experimental glomerulonephritis through macrophage activation (Behmoaras et al., 2008, 2010, 2015) and present a markedly distinct macrophage transcriptome, which results in differential macrophage activation (D'Souza et al., 2013; Maratou et al., 2011). Here, we show that the peripheral monocyte transcriptome of LEW and WKY rats is modulated differentially by the CAF diet. Our results suggest that monocyte transcriptome differences are caused by the contrasting metabolic and morphometric responses of LEW and WKY rats to the diet-induced obesity. These results establish new rodent models of nutritional genomics in CAF-induced obesity.

## RESULTS

### The metabolic response of LEW and WKY rats to CAF diet is strain dependent

We have previously shown in detail strain-specific phenotypic differences in CAF-diet-induced obesity in LEW and WKY rats (Martínez-Micaelo et al., 2016). Strain-specific differences were also observed in the modulation of the circulating levels of inflammatory biomarkers. Although the CAF diet provoked a significant increase in the levels of MCP-1 in LEW rats (241.29±13.0 versus 297.97±11.9, mean±s.d., *n*=5, *P*=0.017), no dietary effects were observed in the circulating MCP-1 levels in WKY rats (273.57±22.0 versus 305.10±10.9, *P*=0.263).

### CAF diet differentially modulates gene expression profiles in circulating monocytes from LEW and WKY rats

**Fig. 1.**
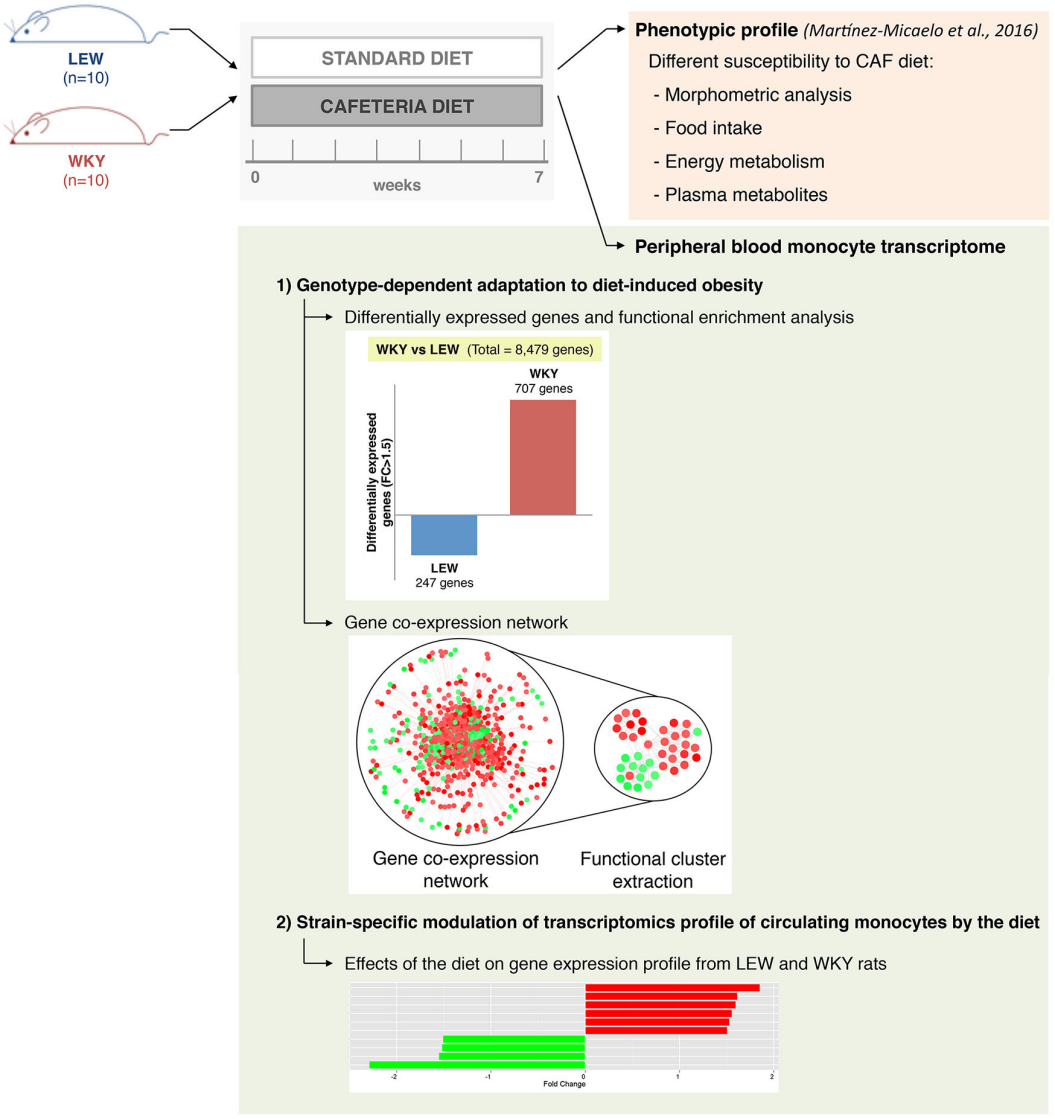
**Experimental design used to study the role of inbred rat models in nutritional genomics.** The phenotypic response (described previously in Martínez-Micaelo et al., 2016) shows a differential susceptibility of LEW and WKY rats to the CAF diet. Here, we studied the differential modulation of the peripheral monocyte transcriptome of LEW and WKY rats by the CAF diet, as consequence of the prone and resistant responses of LEW and WKY rats, respectively, to the diet-induced obesity.

To study genetic factors underlying the metabolic–immunological crosstalk, gene expression profiles of circulating monocytes from standard (STD)-diet- and CAF-fed LEW and WKY rats were determined. Microarray data (deposited at Gene Expression Omnibus under GEO Series accession number GSE85167) were analysed in two ways: (1) comparing the monocyte expression profiles of CAF-fed WKY and LEW rats in order to identify genes that underlie genotype-dependent adaptation to diet-induced obesity, and (2) analysing strain-specific modulation of the transcriptome of circulating monocytes by the CAF diet. The study design is described in Fig. 1.

#### Strain-dependent adaptation of LEW and WKY rats to the CAF diet

**Fig. 2.**
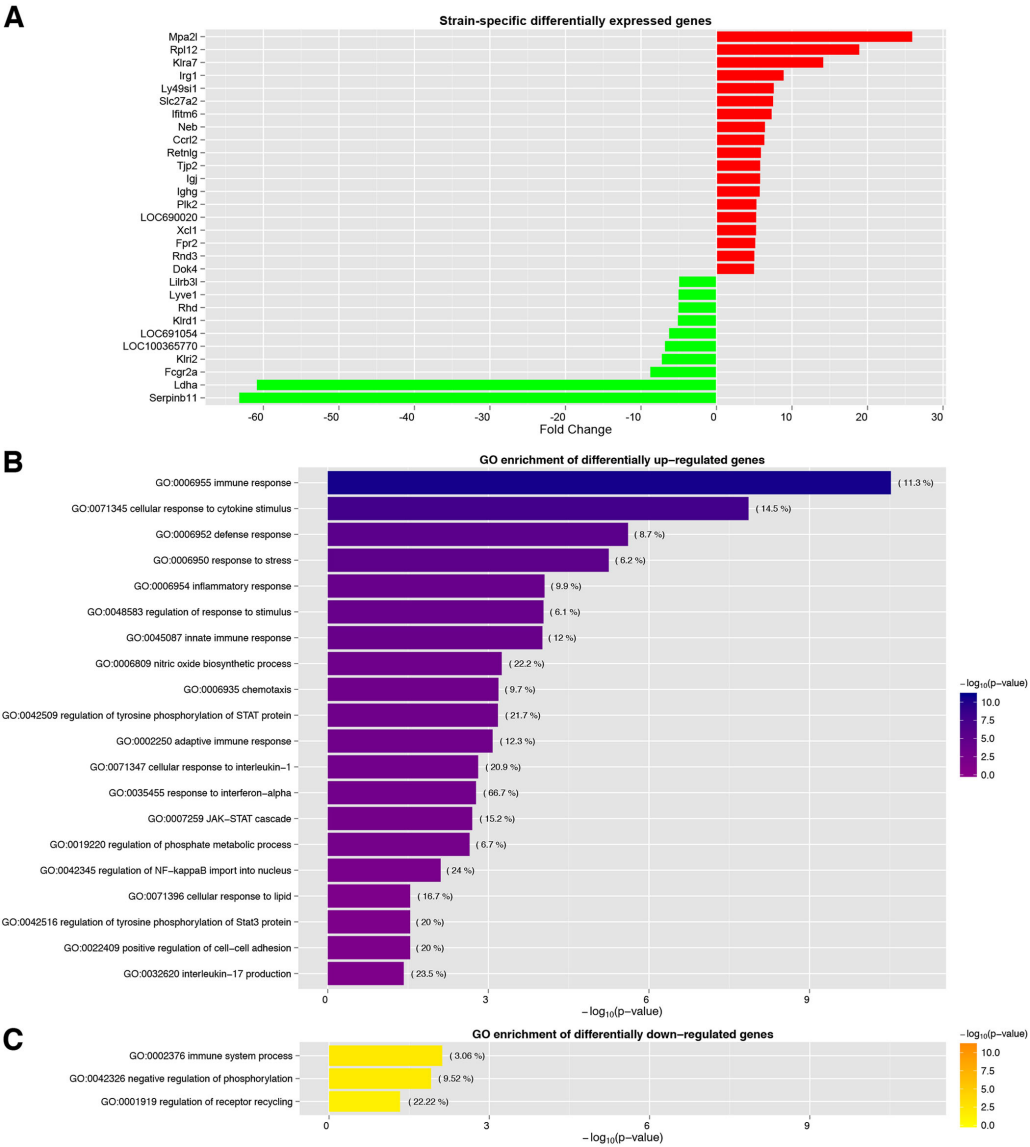
**Gene expression profile in circulating monocytes from CAF-fed LEW and WKY rats.** (A) Strain-specific differentially expressed genes. Transcripts with more than five-fold differences between CAF-fed WKY and LEW rats are shown; the red bars correspond to the transcripts that are overexpressed in WKY rats, and the green bars indicate the transcripts that are overexpressed in LEW rats. (B,C) A summary of the overrepresented GO categories based on a Gene Set Enrichment analysis of the differentially expressed genes between CAF-fed WKY and LEW rats.

The expression profiles of circulating monocytes from CAF-fed WKY and LEW rats were first compared in order to determine the genetic basis of the strain-dependent response to obesity. This microarray data analysis identified 8479 transcripts that were differentially expressed (*P*<0.01); 75.5% of these were significantly upregulated in WKY rats. We prioritised a set of 29 transcripts that were significantly differentially expressed after correction for multiple testing [false discover rate (FDR) <5%] and with a fold change (fc) >5 when comparing the expression profiles from WKY and LEW rats (Fig. 2A). Notably, this subset of highly differentially expressed transcripts included macrophage activation 2 like (*Mpa2l*), a target gene of nuclear factor-κB (NF-κB) (Rothgiesser et al., 2010) and lactate dehydrogenase A (*Ldha*), the gene encoding for the enzyme that catalyses the conversion of lactate into pyruvate during glycolysis under anaerobic conditions. *Mpa2l* was overexpressed by 26-fold in WKY rats, whereas *Ldha* was markedly down-regulated (fc=−60.95) in these rats. Furthermore, six of the 29 transcripts with high differential expression between WKY and LEW rats (*Klra7*, *Irg1*, *Ly49si1*, *Plk2*, *Rnd3* and *Lilrb3l*) had also been previously identified as differentially expressed transcripts in bone marrow-derived macrophages from WKY and LEW rats (Maratou et al., 2011).

To gain insights into the molecular processes and pathways underlying these transcriptional responses, we analysed the set of 954 transcripts with fc >1.5 (up- and down-regulated) between CAF-fed WKY and LEW rats for enrichment for gene ontology (GO) terms. The GO terms were manually curated to remove redundant categories resulting from overlapping gene sets (Table S1), and the most relevant terms that were significantly overrepresented are shown in Fig. 2B. The overexpressed genes in CAF-fed WKY rats were enriched for terms related to the activation of inflammatory and immune responses resulting from the upregulation of classical inflammatory mediators such as interferon γ (*Ifng*, fc=2.43), tumour necrosis factor (*Tnf*, fc=2.11), nitric oxide synthase 2 (*Nos2*, fc=1.68), interleukins (IL) including *IL1a* (fc=2.66), *IL6* (fc=2.41), *IL12a* (fc=2.34), *IL18* (fc=1.96) and *IL23a* (fc=3.45) as well as genes involved in the regulation of eicosanoid production, such as cyclooxygenase 2 (prostaglandin-endoperoxide synthase 2, *Ptgs2*, fc=2.71) and 5-lipoxygenase (arachidonate 5-lipoxygenase, *Alox*, fc=2.70). Moreover, the overexpressed genes were also enriched for terms related to positive regulation of the JAK–STAT cascade, specifically through the involvement of the tyrosine phosphorylation of STAT3. There were very few significantly enriched gene ontologies that were downregulated in CAF-fed WKY rats compared to CAF-fed LEW rats (Fig. 2C), suggesting the prominent significant functional gene enrichment for the immune system GO category.

**Fig. 3.**
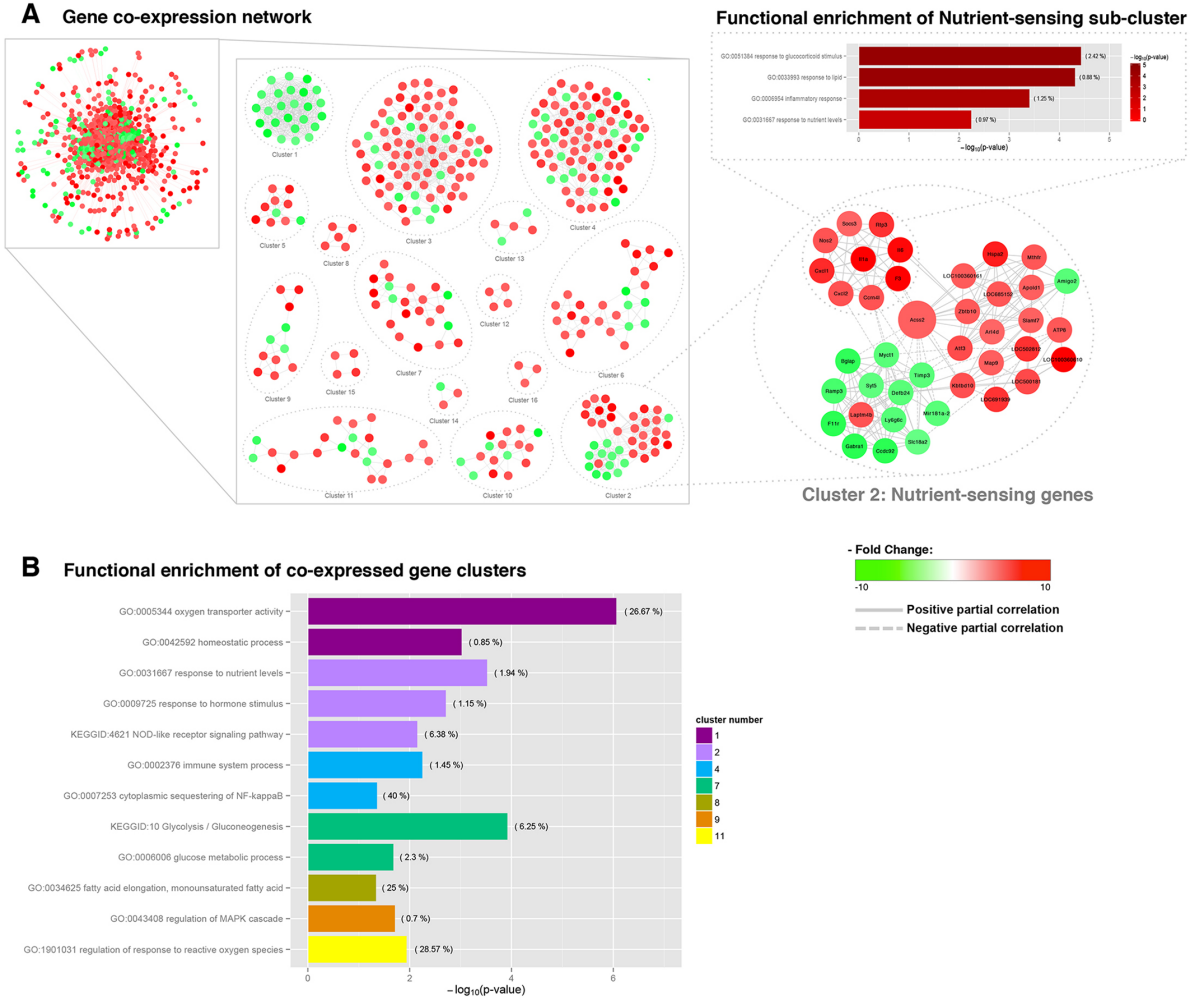
**Gene co-expression networks for differentially expressed transcripts in CAF-fed WKY and LEW rats.** (A) A simplified representation of the network and the MCODE-based clusters. Cluster 2, significantly enriched as a nutrient sensor, is highlighted, and the significantly over-represented GO categories from the selected sub-cluster are shown. The edge shape is related to the direction of the partial correlation; the continuous lines represent partial positive correlations, and dotted lines refer to partial negative correlation. The node colour represents the fold change between the expression of the corresponding transcript in CAF-fed WKY and LEW rats; red nodes correspond to genes that are overexpressed in WKY rats, and green nodes correspond to genes that are overexpressed in LEW rats. The highlighted node within cluster 2 represents a high-scoring differentially expressed gene. (B) Functional enrichment analysis of the MCODE-based co-expressed gene clusters. Significantly over-represented GO terms and KEEG pathways within each cluster are represented.

We then investigated gene regulatory networks by constructing a gene co-expression network based on the analysis of the microarray-derived expression profiles assessed in circulating monocytes. In order to identify the individual genes and gene clusters whose co-regulation underlie strain-specific responses to diet, a gene co-expression network was inferred based on the differentially expressed transcripts that showed a fc >1.5 (up- and down-regulated) between the circulating monocytes from CAF-fed WKY and LEW rats (Fig. 3A). The resulting network is composed of a single connected component formed by 742 nodes and 8215 edges. The molecular complex detection (MCODE) cluster algorithm was employed to extract functional modules of densely interconnected genes (Bader and Hogue, 2003). The biological relevance of the genes included in each of the 16 MCODE-derived clusters (Fig. S1) was tested with GO and Kyoto Encyclopedia of Genes and Genomes (KEGG) analyses (Fig. 3B). Although not all of the modules were enriched significantly for GO terms or KEGG pathways, the transcripts in cluster 2, forming 40 nodes and 205 edges (representing 40 annotated protein-coding genes), were functionally enriched for nutrient sensors (*P*=2.94×10^−4^), responders to hormone stimuli (*P*=1.90×10^−3^), and components of the NOD-like receptor signalling pathway (*P*=6.84×10^−3^). Remarkably, the topology analysis of the module structure of cluster 2 identified acyl-CoA synthetase short-chain family member 2 (*Acss2*) as the most essential hub within the cluster in terms of the node with the largest degree and the highest betweenness score. In addition, the functional relevance of *Acss2* as a regulator of nutrient sensing was determined by analysing the overlap of quantitative trait loci (QTLs) within the *Acss2* genomic region based on previously established QTL mapping results obtained from the Rat Genome Database (de la Cruz et al., 2005). This analysis revealed that a large majority of the 30 overlapping QTLs are related to traits associated with the regulation of metabolism such as body weight QTL 94, glucose level QTL 39 and serum leptin concentration QTL 7 (Table S2).

In order to link the monocyte transcriptome to the obesity phenotypes observed in CAF-diet-fed rats, we investigated the association between the expression levels of *Acss2* in peripheral monocytes and metabolism-related traits (Table 1; Fig. S2). Notably, significant and strong correlations were found between the *Acss2* expression in monocytes and (1) plasma levels of glucose, triglycerides, leptin and non-esterified fatty acids (NEFAs), and (2) total fat and body weight as morphometric parameters (Table 1; Fig. S2).

**Table 1.**
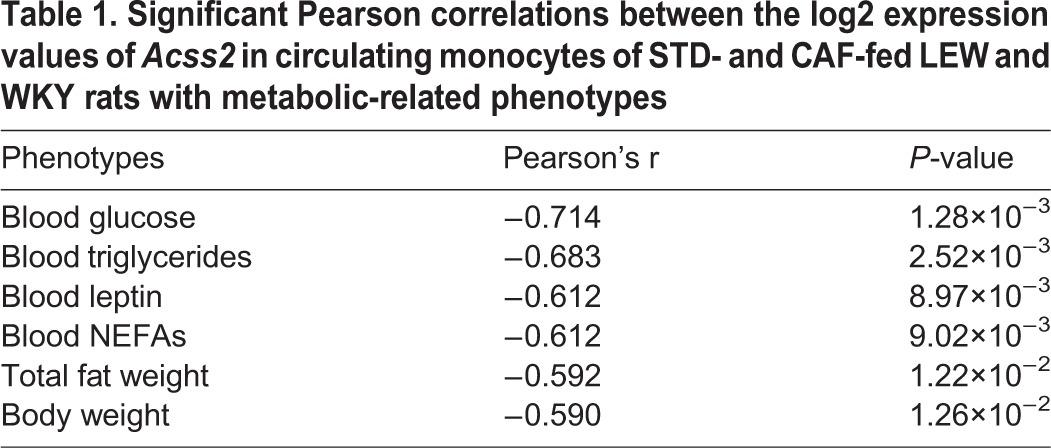
**Significant Pearson correlations between the log2 expression values of *Acss2*** **in circulating monocytes of STD- and CAF-fed LEW and WKY rats with metabolic-related phenotypes**

The topological analysis inside this nutrient-sensing cluster also revealed a functional submodule consisting of nine highly co-expressed nodes; all of these nodes were overexpressed in CAF-fed WKY rats compared to the LEW-fed rats and significantly enriched for inflammatory response genes and genes that function in response to lipid and glucocorticoid stimuli.

#### Strain-specific modulation of the transcriptome by CAF diet in circulating monocytes

**Fig. 4.**
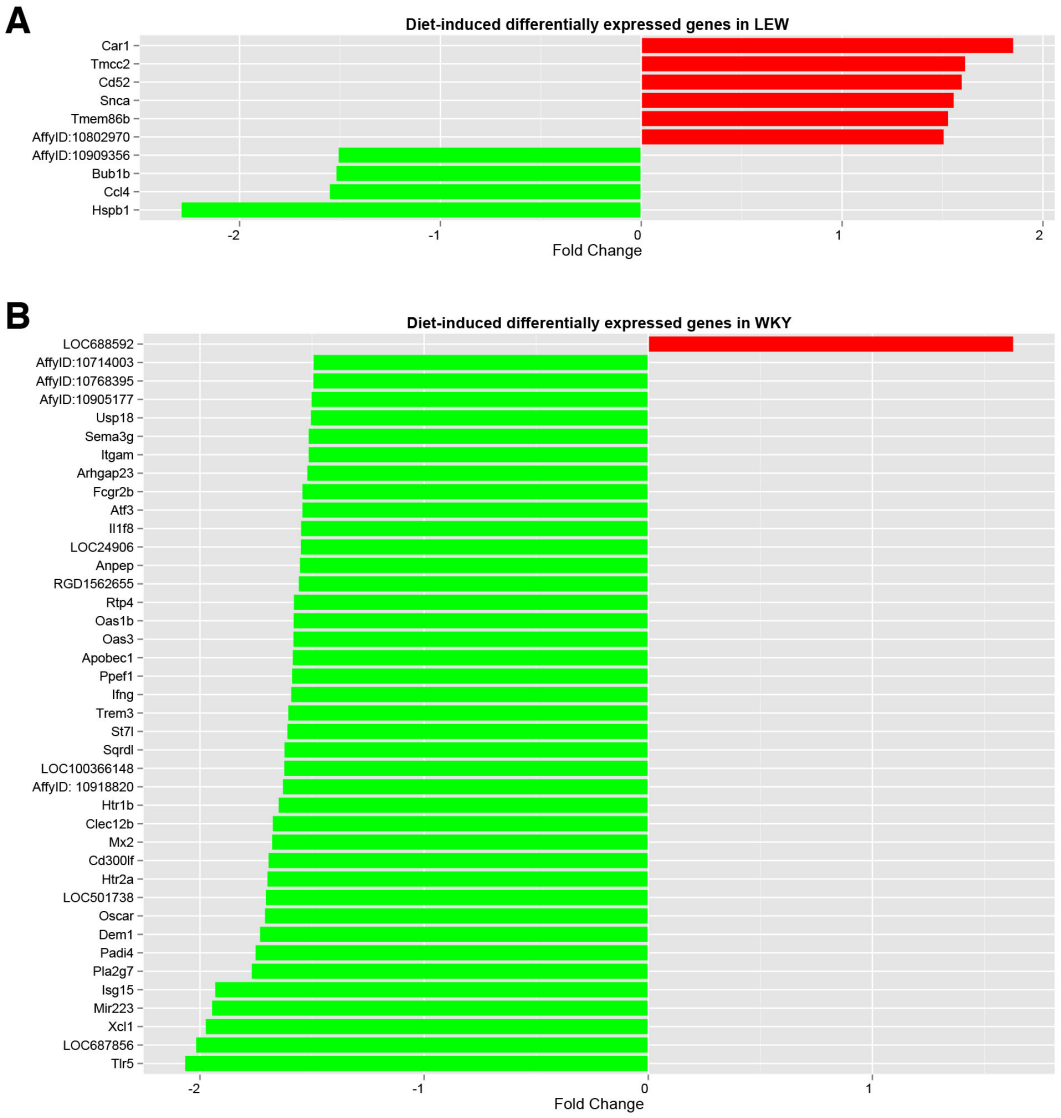
**Effect of** **CAF diet on the expression profile of circulating monocytes from WKY and LEW rats.** (A) The effect of the CAF diet on the transcriptome of circulating monocytes of LEW rats when compared to that of STD diet. (B) The effect of the CAF diet on the gene expression profile of circulating monocytes of WKY rats. Up-regulated and down-regulated transcripts with fold change (fc) >1.5 are shown.

We also determined the effects of CAF diet on monocyte gene expression profile from LEW and WKY rats (Fig. 4A,B). We show that the phenotypic response of LEW rats to CAF diet was translated into significant changes in monocyte gene expression, and although 228 and 195 transcripts were significantly up- and down-regulated respectively, the magnitude of the fold changes induced by CAF diet was relatively moderate. Furthermore, GO and KEGG analysis of the differentially expressed transcripts did not reveal any significant enrichment for any obvious obesity-related pathways when the CAF and STD diets were compared in LEW rats. Interestingly, the modulation of the circulating monocyte expression profile by the CAF diet in the WKY rats resulted in more significant changes in monocyte gene expression levels (483 upregulated and 449 downregulated transcripts). GO and KEGG analyses showed an enrichment for Wnt and peroxisome proliferator-activated receptor (PPAR) signalling pathways for upregulated and downregulated genes, respectively.

## DISCUSSION

In the present study, we show differential monocyte transcriptome responses in inbred rat strains subjected to a metabolic challenge based on dietary energy density. Based on our previous results (Martínez-Micaelo et al., 2016), although both strains showed increased weight gain and adiposity in response to the CAF diet, plasma metabolic profiling evidenced contrasting phenotypic responses. Indeed, CAF diet challenge induced hyperglycaemia, hypertriglyceridaemia and higher levels of NEFAs in LEW rats, which was not the case for WKY rats. These strains are therefore distinct models of diet-induced obesity based on their biochemical response to CAF diet. Furthermore, increased circulating leptin levels were observed independently of the genetic background. In our previous work, using the same model of diet-induced obesity, we reported a strong relationship between plasma leptin levels and the nutritional status-associated traits, such as the food intake, adiposity or plasma triglycerides and glucose levels in LEW rats. The metabolic phenotype resulting from central leptin resistance is considered as a pivotal event underlying the development of obesity and its associated dysfunctions (Zhang and Scarpace, 2006). In contrast, the diet-induced hyperleptinaemia was not translated into the impairment in lipid and glucose metabolisms in WKY rats.

Here, we show that the diet-induced modulation of the inflammatory response is strain specific. Although CAF diet promoted inflammation in LEW rats, in which increased fat-derived circulating MCP-1 levels were observed, this was not the case for WKY rats, suggesting the genetic control of CAF-induced inflammatory markers in plasma.

The genetic determinants of the phenotypic response to diet is well established (Pérusse and Bouchard, 2000; Speakman, 2004). Our results obtained in WKY and LEW rats suggest that the factors underlying the differential response to the dietary energy density are genetically determined in these strains. The role of peripheral monocytes as professional stress-sensing circulating cells suggests that the regulation of gene expression in these cells is crucial for determining host genetic factors influencing the plasticity of the response to diet-induced obesity. Given the contrasting metabolic profiles of WKY and LEW rats, we hypothesised that their monocyte transcriptome will be an intermediate phenotype reflecting the genetic control of CAF diet in the rat. The differentially expressed genes underlying the strain-dependent response to CAF diet were determined by comparing the expression profiles of monocytes from CAF-fed WKY and LEW rats. Notably, the genes that were significantly overexpressed in WKY rats were significantly enriched for the activation of inflammatory and immune response GO categories. Indeed, genes encoding classical inflammatory mediators, including *Ifng*, *Tnf*, *Nos2*, several interleukins and regulators of eicosanoid production were significantly overexpressed in CAF-fed WKY rats.

The measurement of a broader range of inflammatory plasma biomarkers (not only MCP-1 levels) in the two strains will allow more conclusive correlations between the monocyte transcriptome and systemic markers of obesity-induced inflammation. Of note, the expression of Mcp1 is not differentially expressed in response to cafeteria diet in monocytes from WKY and LEW strains, so the levels of this inflammatory biomarker in plasma is not explained by the peripheral monocyte transcriptome, but more likely is a result of adiposity gained during the CAF diet.

A co-expression network of the differentially expressed genes was inferred in order to explore transcriptional changes in terms of gene–gene interactions. The integration of the co-expression network inferred from the significant pairwise partial correlations between the strain-specific genes showing differential expression and the MCODE-based clustering of nodes leads to biological function-enriched clusters of highly co-expressed genes. This is based on the assumption that functionally related genes are frequently co-expressed across organisms, forming conserved transcription modules. Thus, by extracting the cluster of genes differentially co-expressed, we identified a module where highly co-expressed genes were significantly enriched for nutrient sensing, the response to hormone stimuli and NOD-like receptor signalling pathways. Furthermore, *Acss2* was identified as the most highly connected hub gene and also the gene with the highest betweenness centrality score, two topological measures that highlight the impact of this node within the cluster. Based on its position within the cluster, *Acss2* expression changes might have a large impact on the underlying biological functions (Carter et al., 2004). *Acss2* encodes a member of the acyl-CoA synthetase short-chain family, an enzyme that catalyses the ligation of the acetate derived from the β-oxidation of fatty acids to CoA to produce acetyl-CoA, which is further oxidised through the tricarboxylic acid (TCA) cycle. *Acss2* is therefore an essential enzyme for energy expenditure under ketogenic conditions (Luong et al., 2000). QTLs related to complex obesity-related traits such as body weight, glucose and leptin levels were found to overlap within the *Acss2* genomic region. Interestingly, in a previous study investigating glucose-intolerance biomarkers in circulating white blood cells (WBCs) in ApoE3Leiden mice treated with high-fat diet (Wopereis et al., 2012), *Acss2* gene expression was proposed to be an accurate diagnostic biomarker for glucose intolerance. *Acss2* gene expression in WBCs has been linked with hepatic concentrations of TCA-involved metabolites, including citrate, malate, succinate and fumarate, whose production depends directly on the ACSS2 protein activity (Wopereis et al., 2012). Consistent with these results, we found that, in addition to the overexpression of *Acss2* in CAF-fed WKY rat monocytes, which showed significantly lower glucose levels than CAF-fed LEW rats, there is a significant and strong correlation between *Acss2* gene expression in circulating monocytes and the corresponding glucose plasma levels. *Acss2* levels also significantly correlate with plasma leptin levels and the body weight, traits related to the overlapping QTLs, and with the plasma levels of triglycerides and NEFAs. Therefore, our results confirm that a low expression of *Acss2* in circulating monocytes is an indicator of impaired glucose metabolism. Furthermore, *Acss2* is involved in *de novo* lipogenesis, and in the regulation of the triglyceride storage capacity of adipose tissue, playing a key role as a nutrient sensor. In addition, the expression of *Acss2* is regulated by sterol regulatory element-binding proteins (SREBPs), transcription factors, which are key regulators of nutritional homeostasis (Osborne, 2000). SREBPs, and especially SREBP-1, which is abundantly expressed in monocytes, have also been described as a link between the lipid metabolism and the innate immune response through its direct regulation of core genes involved in the inflammasome activation (Im et al., 2011), supporting the significant over-representation of the NOD-like receptor signalling pathway in the co-expressed genes within the *Acss2*-regulated nutrient-sensing cluster. In summary, our results strongly suggest that *Acss2*, a nutrient-sensing protein and a diagnostic biomarker of glucose resistance, is a key regulator gene for the differential responsiveness to dietary energy density and a pivotal genetic link for the integration of metabolic and immune homeostasis.

When CAF-diet effects were compared to those of the STD diet, this translated into a moderate change in gene expression profile in circulating monocytes of the LEW rat, whereas a substantial set of genes was differentially modulated in WKY rats including microRNA 223 (*Mir223*) expression. miR-223, the mature form of *Mir223*, is a microRNA that is highly expressed in monocytes/macrophages and directly targets STAT3 to regulate its activation (Chen et al., 2012).

In conclusion, these results suggest that inbred rat strains could be used in nutritional genomics studies to establish the complex intermediate role of innate immune cells, such as monocytes, in CAF-induced obesity.

## MATERIALS AND METHODS

### Animals and experimental design

Male LEW (LEW/Crl) and WKY (WKY/NCrl) (Charles River, Margate, UK) rats, weighing 190 g and 130 g, respectively, were housed in a 22°C temperature-controlled room with a 12-h-light–12-h-dark cycle. After an adaptation period, rats from each strain were randomly distributed into two experimental groups (*n=*5) and fed with either a standard chow diet (STD) (Panlab, Barcelona, Spain) or a cafeteria diet (CAF), generating a hypercaloric diet distributed in a 10.4%, 38.6% and 50.8% of daily total energy intake from protein, carbohydrates and lipids, respectively. After 7 weeks of the indicated diet, the rats were fasted for 9 h and sacrificed by exsanguination under anaesthesia. Blood was collected from abdominal aorta, and circulating monocytes were immediately isolated. See Fig. 1 and Martínez-Micaelo et al. (2016) for more detailed experimental design. Heparinised plasma was obtained by centrifugation from blood samples and stored at −80°C until analysis. All the procedures were performed with the approval of the Animal Ethics Committee of the Universitat Rovira i Virgili (Tarragona, Spain).

### Measurement of inflammatory parameters in plasma

MCP-1 was measured with an enzymatic colorimetric test kit provided by ABCAM (Cambridge, MA).

### Isolation of circulating monocytes, RNA extraction and microarray preparation

Peripheral blood mononuclear cells (PBMCs) were isolated by density-gradient centrifugation using HISTOPAQUE-1083 solution (SIGMA, Madrid). Monocytes were purified based on their adherence to plastic in serum-free RPMI medium. Then, after 90 min, the non-adherent cells were removed by several washes with warm PBS, and the total RNA from the adherent monocytes was extracted using the TRIzol reagent according to the manufacturer's instructions (Invitrogen, Barcelona, Spain) and purified using RNeasy microkits (Qiagen). The quality of total RNA isolated was determined using a Agilent 2100 Bioanalyser. The RNA Integrity Number (RIN) of RNA ranged from 9.10-10. Total RNA (150 ng) were amplified, labelled and hybridised to Rat Gene 1.0 ST arrays (Affymetrix, Santa Clara, CA) using the Ambion WT Expression kit (Life Technologies) according to manufacturer's instructions.

### Microarray analysis of differential gene expression

The data from microarrays (deposited at Gene Expression Omnibus under GEO Series accession number GSE85167) were normalised using the robust multi-array average (RMA) method (Irizarry et al., 2003), implemented in the Affymetrix Bioconductor package, and the differentially expressed genes were determined using the linear model implemented in the limma Bioconductor package. The Benjamini and Hochberg method was used to adjust *P*-values for multiple testing and control of the false discovery rate. GO terms and KEGG pathways analysis was performed using the GOstats Bioconductor package (Falcon and Gentleman, 2007).

### Gene co-expression networks and statistical analysis

A gene co-expression network was constructed from the gene expression profiles of the transcripts whose expression was differentially modulated (absolute fc >1.5) when comparing the expression profiles of circulating monocytes from the CAF-diet-fed WKY and LEW rats. The co-expression network was inferred using a graphical Gaussian model (GGM) implemented in the R package GeneNet (Schäfer and Strimmer, 2005). Briefly, a partial-correlation matrix was estimated by computing the partial correlation between the expression profiles of each gene pair. Bayesian posterior edge probability >0.95 (corresponding to a local false discovery rate <5%) was used to determine the significance of the resulting pairwise partial correlations. In the resulting co-expression network, the nodes represent the set of genes that were differentially expressed and co-expressed between WKY and LEW rats, and the edges link the pairs of genes whose expression is not conditionally independent, defined as the pairwise partial correlation once the common effects of the other genes in the subset are removed (Opgen-Rhein and Strimmer, 2007). To identify sub-clusters (modules) within the network we used the MCODE algorithm implemented in the clusterMaker plugin (Morris et al., 2011) of Cytoscape software (Cline et al., 2007), and the modules were functionally annotated with GO terms and KEGG pathways using the GOstats Bioconductor package (Falcon and Gentleman, 2007). The selected clusters were analysed using the cyto-Hubba plugin (Lin et al., 2008) to determine the hubs and bottlenecks that represent the key regulatory genes within the network.

### 
